# Unilateral Recurrent Laryngeal and Hypoglossal Nerve Paralysis Following Rhinoplasty: A Case Report and Review of the Literature 

**Published:** 2014-01

**Authors:** Mehdi Bakhshaee, Ali Reza Bameshki, Mohsen Foroughipour, Mohammad Ali Zaringhalam

**Affiliations:** 1 *Sinus and Surgical Endoscopic Research Center, Imam Reza Hospital, School of Medicine, Mashhad University of Medical Sciences, Mashhad, Iran.*; 2 *Cardiac Anesthesia Research Center, Imam Reza Hospital, School of Medicine, Mashhad University of Medical Sciences, Mashhad, Iran.*; 3 *Departments of Neurology, Ghaem Hospital, School of Medicine, Mashhad University of Medical Sciences, Mashhad, Iran.*; 4 *Departments of Otorhinolaryngology, Ghaem Hospital, School of Medicine, Mashhad University of Medical Sciences, Mashhad, Iran.*

**Keywords:** Hypoglossal Nerve, Paralysis, Recurrent Laryngeal Nerve, Rhinoplasty

## Abstract

**Introduction:**

Injury to cranial nerves IX, X, and XII is a known complication of laryngoscopy and intubation. Here we present a patient with concurrent hypoglossal and recurrent laryngeal nerve paralysis after rhinoplasty.

**Case Report:**

The patient was a 27-year-old woman who was candidate for rhinoplastic surgery. The next morning after the operation, the patient complained of dysphonia and a sore throat .7 days after the operation she was still complaining of dysphonia. She underwent a direct laryngoscopy, and right TVC paralysis was observed. Right hypoglossal nerve paralysis was also detected during physical cranial nerve function tests. Hypoglossal and recurrent laryngeal nerve function was completely recovered after 5 and 7 months, respectively, and no complication was remained.

**Conclusion:**

Accurate and atraumatic intubation and extubation, true positioning of the head and neck, delicate and gentle packing of the oropharynx, and maintenance of mean blood pressure at a safe level are appropriate methods to prevent this complication during anesthesia and surgical procedures.

## Introduction

Injury to cranial nerves IX, X, and XII is a known complication of laryngoscopy and intubation. Occurrence of this type of injury has been also reported in association with the use of laryngeal mask airways (LMA) and cuffed oropharyngeal airways (COPA) (1-4). 

In some cases simultaneous involvement of several nerves occurs. Concurrent paralysis of the recurrent laryngeal nerve (a branch of cranial nerve X), and the hypoglossal (XII) nerve is known as Tapia’s syndrome and results in unilateral vocal cord and tongue paralysis. In this study, a patient who underwent rhinoplasty under general anesthesia and intubation complaining of concurrent hypoglossal and recurrent laryngeal nerve paralysis after the operation is presented.

## Case Report

The patient was a 27-year-old woman who weighed 56 kg, ASA I**, **and was a candidate for rhinoplastic surgery. She had no history of disease or previous surgery, her Mallampati score was I, and her mouth opening was normal. Propofol and Phentanyl were used for anesthetic induction, and cisatracurium for induction of muscle relaxation. Following 2 minutes of ventilation with a mask she underwent laryngoscopy and orotracheal intubation. A number 7 tracheal tube was inserted easily, and the cuff inflated with 4 ml air resulting in a pressure of 20 cmH_2_O without any air leakage around the tube. Wet sterile gauze was then inserted into the oropharynx with a laryngoscope and magile forceps to prevent entry of nasopharyngeal secretion into the hypopharynx. The orotracheal tube was fixed in place with a bandage and the patient was positioned in the reverse Trendelenburg position at a 15˚ angle.

Anesthesia was maintained with Propofol, remi-Phentanyl, and 60% N_2_O, and mechanical ventilation was performed using 500 cc volume and 10/min respiratory rate. The patient’s mean blood pressure was reduced to between 50 and 60 mm Hg, but uncontrolled hemodynamic changes and hypoxia or hypercarbia did not occur during the operation. The operation lasted 2 h and 30 min after which the oropharyngeal gauze was removed and the patient was extubated after removing secretions by suction. The patient awoke completely in the recovery room without any complications, so she was transported to the ward after 50 minutes. 

The next morning, the patient complained of dysphonia and a sore throat, which was attributed to the tracheal intubation, and she was reassured that these problems would disappear in the next few days. However, 7 days after the operation she was still complaining of dysphonia. She underwent a direct laryngoscopy and right TVC paralysis was obvious. Right hypo- glossal nerve paralysis was also detected during the physical cranial nerve function tests. Other neurologic examina- tions had normal findings. The patient did not have any sensory-motor disorders or headache, and was referred to a neurologist for supplementary evaluation. A brain MRI, cervical CT scan, and adjunct laboratory tests were performed and all had normal findings. 

The patient’s condition was monitored but there was no improvement in the symptoms for 3 months. However, after this period of time her signs and symptoms began to gradually improve. Hypoglossal and recurrent laryngeal nerve function was completely recovered after 5 and 7 months, respectively, and no complication was remained.

## Discussion

There have been many reports of often unilateral and in some cases bilateral lingual, hypoglossal, and recurrent laryngeal nerve paralysis following endotracheal intubation or LMA insertion(5). Multiple factors have been discussed as the reason for this complication such as extrarotation of the head, traction of the nerves (1), overinflation of the tracheal tube or LMA(5), and difficulty in insertion of the tracheal tube resulting in trauma to the nerves and mucosa (3). However, perhaps the most important cause of cranial nerve trauma during laryngoscopy and intubation is neuropraxia resulting from increased pressure on the nerve from the laryngoscope blade or tracheal cuff (6). 

In particular, the hypoglossal nerve can be injured where it passes the big horn of the hyoid bone^7^ by an LMA or tracheal tube cuff, or by the tip of the laryngoscope blade. The recurrent laryngeal nerve may also be injured in the larynx because of the pressure of the tracheal tube at the posterolateral zone of the thyroid cartilage (7). However, concurrent paralysis of these two nerves (Tapia’s syndrome) is most likely to be related to trauma to the lateral wall of the inferior part of the oropharynx or the superior part of hypopharynx where the two nerves lie beside each other (8). This is the site where the pack is put during rhinoplasty. The pack itself or the pressure caused by it on the endotracheal tube may put pressure on nerves in the surrounding area. All of the potential risk factors related to nerve paralysis may also be intensified during a controlled hypotension especially during a prolonged operation. Also the reverse Trendelenburg position can reduce blood flow in the head and neck below the mean arterial pressure level, which may be an additional risk factor.

It is surprising that 6 of the 11 reports of Tapia’s syndrome (and also our case) have happened after rhinoplasty (7-11). However, rhinoplasty is an operation which has four risk factors for Tapia’s syndrome: intubation and tracheal tube cuff inflation with no peripheral leakage, packing the pharynx, controlled hypotension, and a prolonged operation. Probably in our case all of these factors together caused this complication. Cranial nerve injury after airway management usually appears during the first 24 to 48 hours after the operation and would improve gradually over the following days and months (7). This shows that it occurs through a neuropraxic mechanism because of regional pressure. In our case this mechanism may have been a causal factor too.

## Conclusion

According to the suspected risk factors for cranial nerve injuries during airway management, it seems that accurate and atraumatic intubation and extubation, proper pressure of the tracheal tube cuff, true positioning of the head and neck, delicate and gentle packing of the oropharynx, and maintenance of mean blood pressure at a safe level are appropriate methods to prevent this complication during anesthesia and surgical procedures.
